# Food security and feeding behaviours in low-income, Latinx families with preschool-aged children

**DOI:** 10.1017/S1368980022001884

**Published:** 2022-09-05

**Authors:** Byron A Foster, Deanna Linville, Emma Rose Miller-Bedell, Hannah Mahjoub

**Affiliations:** 1 Department of Pediatrics, Oregon Health and Science University, Portland, OR 97239, USA; 2 School of Public Health, Oregon Health and Science University and Portland State University, Portland, OR, USA; 3 Center for Equity Promotion, University of Oregon, Portland, OR, USA; 4 Department of Pediatrics, Stanford University, Stanford, CA, USA

**Keywords:** Food security, Parent-child relations, Obesity, Children, Latino

## Abstract

**Objective:**

To examine the association between food security and feeding practices in Latinx parents of pre-school-aged children and examine possible effect modification by parental self-efficacy.

**Design:**

Cross-sectional assessment using the US Department of Agriculture screener for food insecurity as the exposure and sub-scales of the Comprehensive Feeding Practices Questionnaire as the outcome with the General Self-Efficacy Scale as an effect modifier. Non-parametric descriptive statistics were used to compare groups based on food security status.

**Setting:**

Two Latinx communities with low-socioeconomic status in Texas in 2017 and in Oregon in 2018–2019.

**Participants:**

Latinx parents of preschool aged children, English and Spanish speaking. Dyads were excluded if they had moderate-severe developmental disabilities, a seizure disorder with a restrictive diet or taking medications known to influence typical growth.

**Results:**

Of the 168 families in Oregon, 65 (38 %) reported food insecurity, and 10 (21 %) of the 48 families in Texas reported food insecurity. Food security was associated with greater parental monitoring practices in both the Texas and Oregon samples. We observed no differences in creating a healthy home food environment by food security status in either sample. Parental general self-efficacy showed evidence of effect modification in Oregon - only parents with lower self-efficacy Keywords showed a significant association between food security and feeding practices.

**Conclusions:**

Latinx parents of preschool children experience high levels of food insecurity, which are associated with maladaptive parental feeding practices. Greater parental general self-efficacy moderates this association and could buffer Children the effects of food insecurity on children’s health.

Nearly one in four Latinx children and adolescents in the United States have obesity^(1,2)^. While the development of obesity is a complex process, there is evidence that obesity in early childhood tracks into adulthood and is more persistent the younger the person is when they develop obesity^(3)^. Food insecurity, defined as limited access to adequate food due to a lack of money or other resources, has been identified as one potential contributor to the early development of obesity. Both food insecurity and obesity are associated with lower socioeconomic status^(4)^. In 2020, 13·8 million households were food insecure (10·5 % of the US civilian population), and 2·9 million households had food insecurity among children. In Latinx families, a disparity is observed with 17·2 %reporting household food insecurity compared with 10·5 % for white families overall^(5)^. As a consequence of the current COVID-19 global health crisis, food insecurity will likely increase as well as associated disparities, and we need an improved understanding of how food insecurity affects health outcomes to help mitigate the risks.

One of the potential mediators of the association between food insecurity and obesity is parental feeding behaviours and beliefs. Parental feeding practices can influence weight status later in adolescence and adulthood^(6,7)^, and there is also evidence that food insecurity can influence parental feeding practices and beliefs^(8)^. The association between food insecurity and feeding practices and the association with childhood obesity among Latinx families is less understood, particularly for families with preschool-aged children. In preschool-aged children, caregivers have a substantial influence on feeding behaviours at this developmental stage *v*. older children who have greater decision-making capacity around food. There is some evidence that an association does exist between parental feeding behaviours, food security and child weight status among Latinx families^(9)^. One study found that Latinx mothers of elementary aged children placed a high value on eating well, and prior work has described some of the strategies used to achieve this, though not explicitly in the context of food insecurity^(10)^. Additionally, parental self-efficacy has been found to influence parental feeding practices, particularly parents’ understanding of how they can change feeding practices^(11)^.

There is a scientific gap in our understanding of how family-level food insecurity affects feeding practices. To address that gap, we examined the association between food insecurity and feeding practices among Latinx families with pre-school-aged children using two independent samples of children from different geographic regions and at different times to examine the consistency of the association. We also examined the role of self-efficacy in moderating how food insecurity may influence feeding practices. Our hypothesis was that food insecurity in Latinx families alters feeding practices for preschool-aged children, and this effect varies by parental self-efficacy.

## Methods

### Sample participants and setting

We analysed data from two study samples of preschool-aged children and their families. Data were collected in Oregon in 2018–2019 and in Texas in 2017. We examined the data collected at each site separately and presented the analyses in parallel given the different study types and selection criteria. The first sample of children for this analysis was drawn from a Latinx population participating in a clinical trial for children with obesity in Oregon – Growing Healthy Together^(12)^. These parent–child dyads (*n* 168) were enrolled in an early education program, Head Start, and two-thirds were from migrant and seasonal farm worker families. Inclusion criteria for this group were: the children had to have obesity defined as ≥ 95th percentile BMI, and be between 2 and 5 years of age. For this sample, food security and feeding behaviour data were collected at baseline and after the intervention at 6 months. For the primary analysis described here, we use the baseline data before any intervention.

The second sample of parent–child dyads (*n* 48) was recruited in San Antonio, Texas as part of a study examining predictors of children’s weight changes^(13)^. Inclusion criteria for this study were having publicly funded health insurance, and they had a range of weights from normal weight to obesity. These children were 2–5 years of age when their growth chart data were abstracted, and with time to recruitment, some were 6 years of age during the study visit.

For both samples, exclusion criteria included moderate-severe developmental disabilities, a seizure disorder, or taking medications known to influence typical growth. In both samples, participants were recruited from an examination of the medical record to screen for eligibility. A phone screen was then conducted to determine interest and eligibility based on ethnicity and the other inclusion and exclusion criteria described above. The surveys were all conducted using a laptop to record responses with a research assistant present to answer questions, and the surveys were available in English and Spanish.

### Measures

We used the same survey methods for both studies with the primary difference being ordering of questionnaires; parents answered all questionnaires. For the exposure variable of food security, we used the Department of Agriculture’s (USDA) six-item, family-level screener for food security^(14)^. We classified children and parents as living in food insecure or food secure households, with scores of 0–1 on the USDA screener classified as food secure and those with values greater than 1 as food insecure. For the outcome of feeding practices, subscales of the Comprehensive Feeding Practices Questionnaire (CFPQ) were administered during the initial enrolment visit^(15)^. Only certain sub-scales were used to reduce respondent burden and because these subscales were most relevant to the behaviours we wanted to examine. Each sub-scale has a five-point range. The monitoring sub-scale (α of 0·78–0·81)^(15)^ consists of four items with higher scores indicating greater tracking of child’s intake of less healthy foods. The control sub-scale consists of five items with higher scores indicating greater parental control of eating behaviours (α of 0·49–0·70)^(15)^, and the home food environment sub-scale consists of four items with higher scores indicating a healthier home food environment (α of 0·75)^(15)^. We measured self-efficacy using the General Self-Efficacy Scale (GSES), validated in English (α range from 0·76–0·90)^(16)^ and Spanish (α range from 0·77– 0·86)^(16)^. This is a ten-item scale with each item having a four-point range. The Behavioral Risk Factor Surveillance System Questionnaire was used to gather demographic covariate characteristics including parental sex, race/ethnicity, household size, income, employment and education. Income was scaled to the federal poverty line after accounting for family size. Parental height and weight were measured in duplicate with the average recorded.

### Analytic plan

**Fig. 1:**
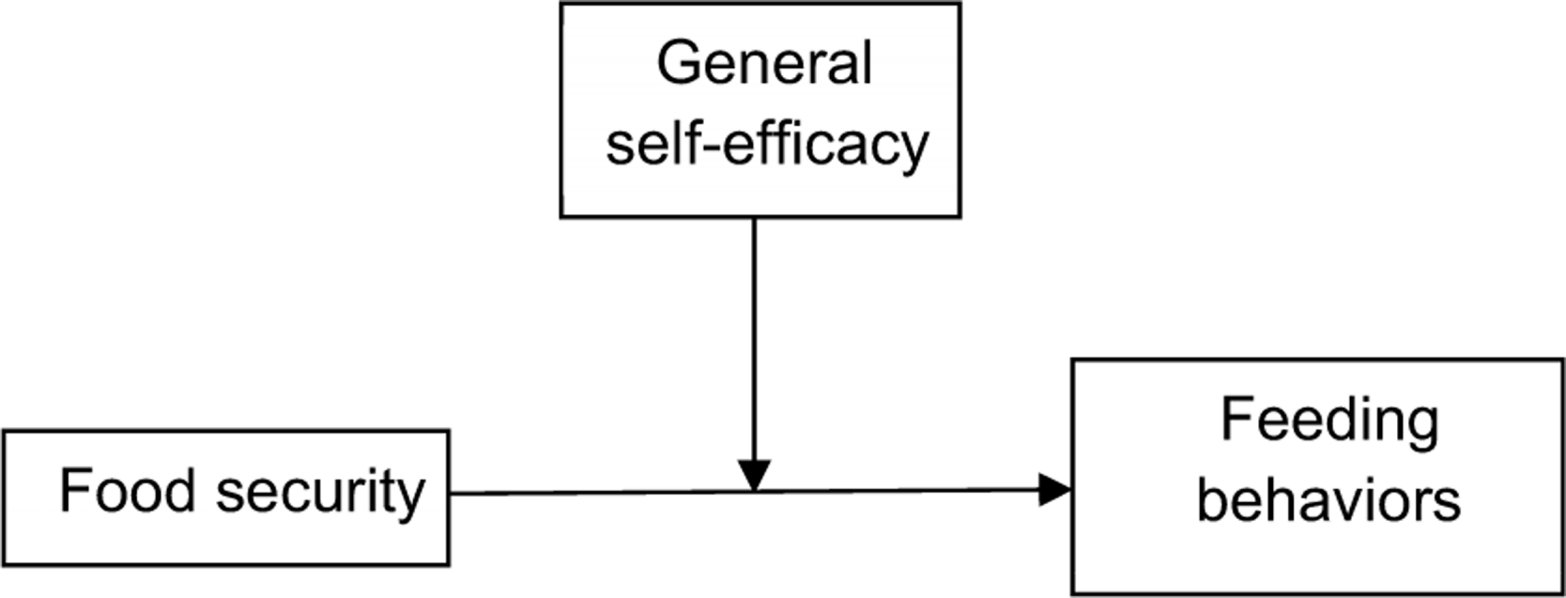
Conceptual model of general self-efficacy (GSES) as an effect modifier of the association between food security and feeding behaviours

We classified children and parents by the primary exposure of family-level food security. The outcome variables of the CFPQ sub-scales were examined for normality using the Shapiro–Wilk test. Non-parametric statistics (Mann–Whitney U test) were used to compare samples by exposure status for each of the CFPQ outcomes; Chi-squared tests were used to examine categorical variables; *t* tests were used to compare continuous variables approximating normal distributions. Linear regression analyses were also examined with the dependent variable of CFPQ outcomes and the independent variable of food security, adjusting for the child’s age and biological sex. We used Spearman’s correlation coefficient to examine the association between the self-efficacy measure (GSES) and feeding behaviours (CFPQ). SPSS v. 27 (IBM) was used for all analyses, and we used a type 1 error rate of 0·05 to determine significance. We also examined whether self-efficacy acted as an effect modifier of the relationship between food security and feeding behaviours by examining the association in those with high self-efficacy (top two quartiles) *v*. low self-efficacy (bottom two quartiles) (Fig. 1).

## Results

In the Oregon sample, families were Latinx and largely low-income and 65 (39 %) of the 168 families reported food insecurity (Table 1). Parents in Oregon had a mean BMI in the obese range for adults. In the Oregon participants, household food insecurity was associated with lower parental education. We found that food secure families reported higher monitoring, with a median of 4·5 (interquartile range (IQR) 4·0, 5·0) compared to food insecure families, median of 4·0 (IQR 3·1, 4·9), *P* = 0·03 (Table 2).

In the Oregon sample, greater self-efficacy was positively correlated with higher scores on the monitoring scale (Spearman’s coefficient of 0·16, *P* = 0·03), negatively correlated with the control scale (Spearman’s coefficient of – 0·20, *P* = 0·007) and positively correlated with the healthy food environment scale (Spearman’s coefficient of 0·33, *P* < 0·001).

We examined whether self-efficacy may be an effect modifier of the association between food security and feeding behaviour (Table 3). We found that only those with lower self-efficacy, defined as having scores in the bottom two quartiles in the sample, had a significant association between food security and the monitoring sub-scale. Of parents with lower self-efficacy, those with food security had a median monitoring score of 4·4 (IQR 3·5, 5·0) compared to those with food insecurity who had a median score of 3·8 (IQR 3·0, 4·5), *P* = 0·03, Mann–Whitney U test. In those in the higher two quartiles of self-efficacy, there was no significant association between food security and monitoring, *P* = 0·23. For parents with lower self-efficacy in the Oregon sample, there was no association between food security and the control sub-scale, *P* = 0·95. In contrast, there was a significant association between food security and controlling feeding practices in those with higher self-efficacy; those with food security had a median score of 2·6 (IQR 2·0, 3·3) compared with those with food insecurity, median 2·9 (IQR 2·6, 3·8), *P* = 0·04. We found no significant differences according to self-efficacy for the healthy home food environment sub-scale.

For the Texas sample, 10 (21 %) of the 48 families reported food insecurity (Table 1). In the Texas participants, we found that monitoring was higher in the food secure sample, median 4·4 (IQR 3·4, 5·0), compared to those with food insecurity, median 2·8 (IQR 2·0, 3·3). Parental controlling feeding practices were higher in the food insecure group in the Oregon sample, and there was no significant difference in healthy food environment scores (Table 2). In the Texas sample with a much smaller number of participants, self-efficacy was not significantly correlated with any of the CFPQ scales (data not shown).

## Discussion

This study examined the association between food insecurity and feeding practices in two Latinx populations, one in Texas and one in Oregon. We found that in these samples of Latinx families from low-socioeconomic backgrounds, caregivers reporting food insecurity were significantly less likely to monitor their child’s consumption of unhealthy food and more likely to report controlling feeding behaviours compared to food secure caregivers. Na et al. observed that Head Start parents with low food insecurity reported less monitoring and that the association is attenuated by food resource management^(17)^. Our study supports the findings that greater food security status is associated with healthier parental feeding practices.

In our analysis, we also found some evidence of effect modification by parental self-efficacy. Parents with lower self-efficacy who experience food insecurity did report more maladaptive feeding behaviours. In parents with higher self-efficacy, food insecurity was not associated with differences in feeding behaviours in these samples. In this analysis overall, there was no association between food insecurity and self-efficacy whereas prior studies have demonstrated that a higher rating of self-efficacy is associated with lower levels of food insecurity^(18–20)^. One possible explanation is sample size – all three of these studies had 200 or more participants^(18–20)^, and our findings in a smaller sample showed a potential trend towards higher self-efficacy in those with food security. Another difference may be context – the studies examining this previously did so with adults at food banks or at a clinic, and we had relatively higher self-efficacy in this sample compared to those prior studies.

Self-efficacy refers to one’s confidence in their ability to change behaviours. The findings described here are consistent with prior studies showing that self-efficacy relates to the ability to change feeding practices. Other researchers have also found that higher parental self-efficacy is associated with healthier feeding practices^(21,22)^. Previous studies found a positive association with self-efficacy and parental monitoring of a child’s diet and less restrictive parenting^(21,23)^. Others have found self-efficacy to be positively associated with higher nutritional quality of diet, including a higher intake of fruit and vegetables^(24)^. The findings of this study and previous ones identify self-efficacy as a potential target to reduce food insecurity, improve quality of diet and improve feeding practices for caregivers and children.

Less parental monitoring of a child’s food intake has critical ramifications on a child’s current and future health^(25)^. Parental monitoring is positively associated with healthier behaviours, such as lower consumption of unhealthy foods and sugar-sweetened drinks^(25)^, and monitoring of dietary fat intake is associated with reduced child BMI *z*-scores^(26)^. Positive reinforcement of healthy behaviors and monitoring children’s dietary intake and physical activity are positively associated with healthy eating and exercise, while controlling, or restrictive parenting is associated with less healthy eating and more sedentary behaviours^(27)^.

In the context of the pandemic, a recent study examined changes in food security status over time. Both food insecurity and parental monitoring increased during COVID-19, and they did not observe an association between changes in food security status and monitoring^(28)^. The COVID-19 pandemic has highlighted the urgency of identifying strategies to address the increasing rate of food insecurity and associated negative feeding patterns.

Limitations of the study include the reliance on self-reporting from participants with the potential for social desirability bias and the cross-sectional study design. All of the children included in the Oregon sample had obesity as did most of the children in the Texas sample. The relationship between food security and feeding behaviours may be different in samples of normal weight or in underweight children. We measured general self-efficacy rather than self-efficacy specific to dietary or feeding behaviours. These scales have been developed and should be investigated in understanding how food security and feeding practices are associated^(29)^.

Despite these limitations, the present study provides a foundation upon which future projects can be conducted to examine the relationships between self-efficacy, parental feeding patterns and food security. This study addresses a gap in the current literature about food insecurity and feeding behaviours in Latinx families and provides insight into potential strategies in designing successful interventions.

## Conclusion

This study found that food insecurity in Latinx families from low socioeconomic status backgrounds is associated with less parental monitoring and higher controlling behaviours related to their child’s food intake, underscoring the complex nature of the impact of food insecurity on childhood health and illness. Our findings showing the potential effect modification of self-efficacy on feeding practices in concert with others’ findings showing that food resource management may mitigate the effects of food insecurity suggest that addressing parental self-efficacy while also supporting access to healthy foods may be a viable intervention strategy to influence feeding behaviours.

## Figures and Tables

**Table 1:** Demographic and anthropometric characteristics of participants recruited from Oregon and Texas research studies, presented separately

	Food secure (*n* 141)		Food insecure (*n* 75)		
	Mean	SD	Mean	SD	*P*-value
Oregon – children	(*n* 103)		(*n* 65)		
Age in years	4·4	0·7	4·3	0·8	0·57
Gender					
*n*	47	30		0·95	
% female	46		46		
BMI z-score	2·3	0·7	2·3	0·7	0·94
BMI percentile	97	5	98	4	0·65
Oregon – parents					
Age in years	33	7	34	7	0·25
Gender					
*n*	99		63		0·70
% female	96		97		
BMI	33	7	34	7	0·19
People in household	5·1	1·7	5·3	1·5	0·46
	*n*	%	*n*	%	
Parental education					0·02
Less than high school	55	53	49	75	
High school and some college	44	43	15	23	
College graduate or more	4	4	1	2	
Income as % FPL					0·85
≤ 100 % FPL	79	77	52	80	
> 100 FPL	5	5	3	5	
Not known/refused	18	18	10	15	
	Mean	SD	Mean	SD	
Texas – children	(*n* 38)		(*n* 10)		
Age in years	6·0	1·4	5·5	0·7	0·25
Gender					
*n*	18		5		0·88
% female	47		50		
BMI z-score	1·9	0·8	1·7	0·6	0·46
BMI percentile	93	10	92	13	0·81
Texas – parents					
Age in years	36	10	34	7	0·55
Gender					
*n*	29		18		0·43
% female	97		100		
BMI	32	7	35	8	0·26
	*n*	%	*n*	%	
Parental education					0·25
Less than high school	16	42	7	70	
High school diploma or equivalent	19	50	3	30	
Any college or college graduate	3	8	0	0	
Income as % FPL					0·22
≤ 100 % FPL	27	71	9	90	
> 100 FPL	9	24	0		
Not known/not sure	2	5	1	10	

FPL = federal poverty line.

All comparisons done using Mann–Whitney U tests for non-parametric data; *t* tests were used for data approximating a normal distribution; Chi-square tests for categorical data.

**Table 2:** Food security, comprehensive feeding practice questionnaire scales and general self-efficacy scale (GSES)

	Food secure (*n* 103)		Food insecure (*n* 65)		
Oregon sample	Median	IQR	Median	IQR	*P*-value
Parental monitoring of less healthy foods	4.5	4.0, 5.0	4.0	3.1, 4.9	0.03
Child control of eating behaviors	2.6	2.2, 3.4	3.0	2.6, 3.4	0.09
Healthy Home food environment	4.0	3.5, 4.5	4.3	3.8, 4.5	0.29
Self-efficacy (GSES)	4.5	4.2, 4.8	4.3	4.0, 4.8	0.16
Texas sample	(*n* 38)		(*n* 10)		
Parental monitoring of less healthy foods	4.4	3.4, 5.0	2.8	2.0, 3.3	0.007
Child control of eating behaviours	2.4	1.8, 2.8	2.2	1.8, 2.3	0.57
Healthy food environment	4.5	4.0, 5.0	4.0	3.1, 4.4	0.07
Self-efficacy (GSES)	4.5	4.0, 4.9	4.3	3.4, 4.7	0.36

IQR = interquartile range; GSES = General self-efficacy scale.

All comparisons done using Mann–Whitney U tests for non-parametric data.

**Table 3:** Stratification by level of general self-efficacy (GSES) to examine for effect modification of the relationship between food security and feeding practices, results shown for the Oregon sample only

Oregon sample	Food secure		Food insecure		*P*-value
Lower self-efficacy (below median)					
Parental monitoring of less healthy foods	4.4	3.5, 5.0	3.8	3.0, 4.5	0.03
Child control of eating behaviors	3.0	2.6, 3.6	3.0	2.6, 3–4	0.95
Healthy Home food environment	3.9	3.5, 4.3	3.8	3.3, 4.3	0.94
Higher self-efficacy (above median)					
Parental monitoring of less healthy foods	4.5	4.0, 5.0	4.5	3.3, 5.0	0.23
Child control of eating behaviours	2.6	2.0, 3.3	2.9	2.6, 3.8	0.04
Healthy food environment	4.0	3.5, 4.5	4.3	4.0, 4.8	0.11

IQR = interquartile range; GSES = General self-efficacy scale.

All comparisons done using Mann–Whitney U tests for non-parametric data.
